# An Empirical Study of the Behaviors of Korean Golf Travelers Based on the Choice Attributes of Golf Courses in Southeast Asia

**DOI:** 10.3390/ijerph19148648

**Published:** 2022-07-15

**Authors:** Sheng-Yen Lee, Ryang-Suk Lee

**Affiliations:** 1 Department of Human Health Care, Gyeongsang National University, 33 Dongjin-ro, Jinju-si 52725, Gyeongsangnam-do, Korea; sylee7@gnu.ac.kr; 2 Sejong University, 209 Neungdong-ro, Gwangjin-gu, Seoul 05006, Korea

**Keywords:** golf travel, sports tourism, golfer’s behaviors, choice attributes, golf course management, Southeast Asia, mixed methods research

## Abstract

The purpose of this study was to understand customer behavior among Korean golf travelers based on the choice attributes of golf courses in Southeast Asia. This study was based on Creswell and Clark’s triangulation design, a mixed-methods research framework that compares the results of quantitative and qualitative investigations. The results of the quantitative study were as follows. ‘H1. Golf course choice attributes will have a positive effect on customer satisfaction’ was partially accepted. Among choice attributes, course management, price, operations management, and lodgings had an effect on customer satisfaction. ‘H2. Customer satisfaction will have a positive effect on intention to revisit’ was accepted. ‘H3. Customer satisfaction will have a mediation effect on the relationship between intention to revisit, and golf course choice attributes’ was partially accepted. Among choice attributes, customer satisfaction only showed a mediating effect in the relationship of intention to revisit with course management and price. The novelty of this study is that we performed mixed-methods research, which has not been carried out in previous studies. Furthermore, we conducted in-depth interviews only with golfers who visited courses in South Asia, selected based on their duration and purpose of the visit. The results of the qualitative study were compared with those of the quantitative study to provide empirical evidence that can be used to help domestic golf travel industry and golf courses in Southeast Asia.

## 1. Introduction

Korean sports have grown rapidly, keeping pace with the nation’s meteoric economic development [1]. When income improves because of economic growth, people start pursuing a better quality of life and show greatly increased interest in health and wellbeing [2,3,4]. The number of people participating in leisure activities has continuously increased, seeking self-development and happiness through physical activity [5].

For modern individuals pursuing better leisure activities in an advanced economy, golf has been highlighted as an environmentally friendly, participatory sport [6,7]. Golf provides pleasure and entertainment and is a challenge in the form of improving one’s score. Golf has become a popular leisure sport due to simultaneously fulfilling self-actualization and social networking needs [8]. Along with being a participatory sport, the continued efforts of competitive Korean golfers domestically and overseas have helped to establish golf as a spectator sport. Recently, the appeal of golf simulations has accelerated its popularization.

As shown in Table 1, the domestic golfing population increased from 2.75 million people in 2007 to 7.61 million people in 2017, and annual golf expenditure during the same period approximately doubled [9]. Meanwhile, 573 golf courses are in operation as of January 2019, including 467 full courses and 104 courses of 10 holes or less [10], representing an almost threefold increase compared with the early 2000s [11].

With increased activity in the domestic golf market, the scale of overseas golf travel has also shown persistent growth. The number of people participating in overseas golf travel increased nearly four times from 2007 to 2017. This increase can be attributed to several factors limiting the domestic golf industry, such as the expensive fees, the unfavorable conditions in winter, and matches being disrupted by the large numbers of guests [12]. The revolutionary growth and popularization of the golf industry has attracted many people to golf. Hence, even compared with neighboring Japan, green fees in South Korea were already around three times more expensive in 2007 [13].

According to statistical data from the 2017 Korean Golf Index, the top reason for overseas golf travel was “to enjoy golf in winter” (23.3%), followed by “inexpensive cost” (18.9%), “friends/golfing groups/other groups” (16.8%), and “to play comfortably” (11.7%) [10]. This provides statistical evidence of increase in overseas golf travel due to the limiting factors of climate and cost in domestic golf. In a 2017 survey of golf travel destinations (Table 2), Southeast Asian countries dominated the rankings, accounting for 57.6% of the responses [9]. Southeast Asian countries are popular as the most appropriate overseas golf travel destinations because of much cheaper prices compared to domestic golf courses; the lower numbers of guests, which allows enjoying a relaxing round of golf without crowding; and the climate, which remains suitable for golf all year round, without seasonal restrictions [14]. Furthermore, Lee and Lee [1] noted an extremely intense competition between golf courses in Southeast Asia and nearby China and Japan to attract Korean golfers. Thus, the 22% decrease in the mean overseas golf travel expenses per person from 2007 to 2017 can be attributed to pricing competition among domestic golf package travel agencies and overseas golf courses [9].

Cooper and Boniface [15] suggested various factors, including amenities, accessibility, and maintenance, to be considered when selecting a leisure facility, such as a golf course. Different properties of a given facility influence choice and assessment. Users compare various properties with similar competing products to choose the product that best suits their needs [16]. Because usage is determined by choice attributes, Lancaster [17] identified choice attributes as the most important elements for selecting a product.

Overseas golf travel includes the major choice attributes of regular golf courses, such as the course itself, course management, round management, practice facilities, and club houses, and travel-related choice attributes, such as accommodation, meals, and airport pickup services. The overseas golf travel can be categorized based on the aims and duration of travel, including short golf tours (social gatherings, family holidays, relaxation, and tourism), mid-to-long-term golf travel by the general public, professional golfers traveling for practice, and long-term stays by retirees. Improving competitiveness to meet the needs of different types of guests is important for survival. In the overseas golf travel sector, specialization is essential to match guests’ objectives and length of stay. Therefore, it is important to target development to satisfy the preferences, in terms of price and objectives, of specific consumers, and to appeal to users through choice attributes [18].

Most studies in this field are limited to the choice attributes of golf courses themselves, rather than choice attributes related to golf travel [19,20,21,22,23,24]. These studies divide the choice attributes of golf courses into factors such as price, facilities, and services, but do not differentiate between different types of visitors. Existing studies on overseas golf travel [12,14] examined limiting factors and perceived value for golf travelers. There has been almost no research on choice factors for overseas golf travel. Considering that most cases of overseas golf travel from Korea are visits to golf courses in Southeast Asia, we can readily infer that the preference factors will differ from golf tours in Japan, the US, or Oceania, which are in a different price bracket than the golf courses in Southeast Asia. Given the current saturation of competition, failure to provide services suited to the duration and objectives of golf travel will inevitably lead to loss of profitability due to price competition for all domestic travel agents offering golf packages and all golf courses in Southeast Asia. Therefore, in this study, we surveyed Korean golfers and conducted a comprehensive analysis of the choice attributes of Southeast Asian golf courses, customer satisfaction, and intention to revisit. In this way, we aimed to identify the choice factors with the greatest effects on customer satisfaction and intention to revisit, and to compare the major choice factors between different types of golfers and different travel objectives.

This study is different from previous research because we only included golfers who visited golf courses in Southeast Asia. We performed mixed-methods research, conducting in-depth interviews with participants selected based on their duration and purpose of visit. The results of the qualitative study were compared with those of the quantitative study to provide empirical evidence that can be used to help domestic golf travel industry and golf courses in Southeast Asia.

## 2. Theoretical Background

### 2.1. Choice Attributes for Overseas Golf Courses

Choice attributes for overseas golf courses include various factors, including the original purpose of golf; additional facilities, such as meals, accommodation, and practice; and travel-related factors, such as visit duration and tourism and travel near the golf course. Travelers choose a golf course based on a comparative evaluation based on different elements constituting the golf course itself as well as the different motivations for visiting overseas golf courses. Given the vast number of diverse attributes of golf courses, the choice of golf course is a complex process involving multiple attributes [25]. Attributes are tangible and intangible characteristics of an object, and users compare various characteristics to select products and services that fit their needs [16]. Thus, choice attributes can be considered key elements for selecting products, and travelers can decide whether or not to visit a place depending on the quality of choice attributes [17]. The choice attributes of golf courses can be thought of as the human and physical environments that constitute the course. Users select a course suited to their purpose by reviewing and comparing these choice attributes. This means that it is important to see whether user interest and appeal can be improved via choice attributes [18]. Higher prices can lead to increased expectations for a product. Because golf is relatively expensive compared with other leisure activities, expectations are high for choice attributes such as customer service [26]. Meeting these expectations can lead to satisfied customers, but failure to meet expectations can result in more negative ratings.

### 2.2. Customer Satisfaction

Discrepancy may exist between customers’ predictions or expectations of a product, based on information obtained before purchase or use, and customers’ feelings or evaluation of the results after actually using the product [27]. Positive feelings or perceptions resulting from the gap between prior expectations and actual experience can be described as satisfaction [28,29]. When users rate their personal experiences with a product, satisfaction can be considered a positive rating [30]. The concept of customer satisfaction can be explained by the expectation disconfirmation theory. Oliver [25] reported that satisfaction occurs because of incongruence between expectations and actual experience. Users form expectations about a product before purchase, and then compare their actual experience after purchase with these expectations. Negative disconfirmation occurs when the experience falls short of expectations, positive disconfirmation occurs when the product exceeds expectations, and simple confirmation occurs when expectations and experience are similar. Thus, when users’ actual experience is better than expected, this results in positive disconfirmation, which manifests as satisfaction. Conversely, expectations higher than the actual experienced results are associated with dissatisfaction. Satisfaction requires that the benefit provided to the user is greater than the cost spent by the user.

In previous research [31] when factors affecting satisfaction of golf course users were investigated, physical factors and accessibility showed positive effects on satisfaction. Good course composition and management, conveniently accessible land, and ease of use of relevant information have been identified as factors related to satisfaction. Cronin and Taylor [32] and Bitner [33] investigated the relationship between customer satisfaction and human factors such as service. Intangible factors such as in-person service affected satisfaction, and satisfaction could be linked to repurchase. Previous studies have demonstrated that choice attributes and satisfaction can be related, but these have tested only unidimensional attributes. Further studies on the specific effects of diverse choice attributes are required.

### 2.3. Intention to Revisit

The intention to revisit reflects the users’ subjective intention to repeatedly use the same product or service. Therefore, it primarily gives an indication of the likelihood of purchasing and can be used to predict future customer behavior, such as positive word-of-mouth [34]. Positive word of mouth and satisfaction are major factors for inducing revisits. A satisfactory experience can be the basis for the relationship between continued purchases and use [35]. Customer’s satisfactory experience of use can lead to positive behaviors related to the product [36]. The positive perceptions formed through experience of use are linked to revisits or repurchases. When the customer shows an intention to revisit, psychological bonding and familiarity for the product increase, which can develop into a future intention to recommend [37]. Among choice attributes for a product, physical and intangible aspects, such as service, play relatively important roles in satisfaction and intention to revisit [34,38]. Jones and Sasser [39] used the intention to revisit to rate customer loyalty. When a customer develops an intention to revisit based on a satisfactory experience and this experience is repeated, it can lead to loyalty. This suggests that satisfaction and intention to revisit are mutually related.

## 3. Methods

### 3.1. Study Design

This study empirically analyzes customer behavior among Korean golf travelers according to the choice attributes of golf courses in Southeast Asia. Among mixed-methods research, the triangulation design of Creswell and Clark [40] was chosen for this study, which compares the results of a quantitative investigation and a qualitative investigation.

### 3.2. Quantitative Study

#### 3.2.1. Hypothesis Setting and Research Model

**Figure 1:**
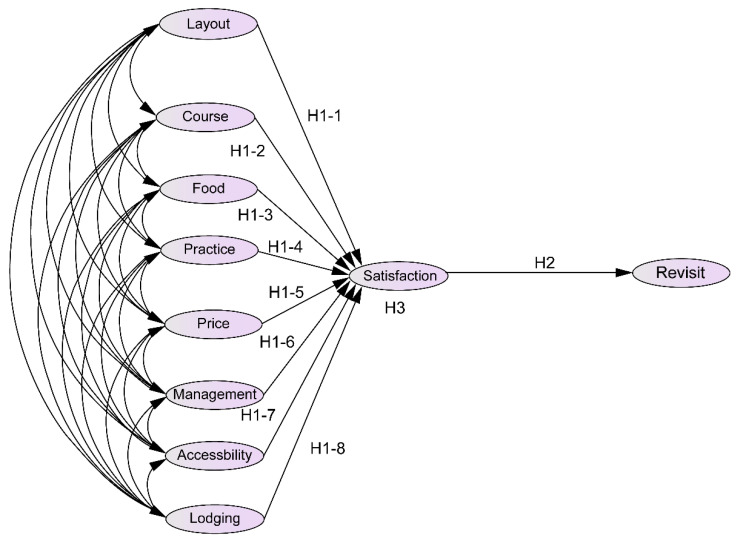
Research model.

In the quantitative part of this study, we examined the relationships of choice attributes of golf courses in Southeast Asia (layout, course, food, practice facilities, price, management, accessibility, lodgings) with customer satisfaction and intention to revisit (Figure 1). We investigated the role of customer satisfaction in the relationship between choice attributes and intention to revisit. Based on previous studies and in accordance with the objectives of this study, we set the following hypotheses:

  **H1.**  *Golf course choice attributes have a positive effect on customer satisfaction*.
    **H1-1.**  *Course layout has a positive effect on customer satisfaction*.
    **H1-2.**  *Course management has a positive effect on customer satisfaction*.
    **H1-3.**  *Food has a positive effect on customer satisfaction*.
    **H1-4.**  *Practice facilities have a positive effect on customer satisfaction*.
    **H1-5.**  *Price has a positive effect on customer satisfaction*.
    **H1-6.**  *Operations management has a positive effect on customer satisfaction*.
    **H1-7.**  *Accessibility has a positive effect on customer satisfaction*.
    **H1-8.**  *Lodgings has a positive effect on customer satisfaction*.
  **H2.**  *Customer satisfaction has a positive effect on intention to revisit*.
  **H3.**  *Customer satisfaction has a mediation effect on the relationship between intention to revisit and golf course choice attributes*.
    **H3-1.**  *Customer satisfaction has a mediation effect on the relationship between intention to revisit and course layout*.
    **H3-2.**  *Customer satisfaction has a mediation effect on the relationship between intention to revisit and course management*.
    **H3-3.**  *Customer satisfaction has a mediation effect on the relationship between the intention to revisit and food*.
    **H3-4.**  *Customer satisfaction has a mediation effect on the relationship between intention to revisit and practice facilities*.
    **H3-5.**  *Customer satisfaction has a mediation effect on the relationship between intention to revisit and price*.
    **H3-6.**  *Customer satisfaction has a mediation effect on the relationship between intention to revisit and operations management*.
    **H3-7.**  *Customer satisfaction has a mediation effect on the relationship between intention to revisit and accessibility*.
    **H3-8.**  *Customer satisfaction has a mediation effect on the relationship between intention to revisit and lodgings*.

#### 3.2.2. Survey Methods and General Characteristics of Subjects

For the quantitative study, focusing on golf courses and practice facilities in the Seoul and Gyeonggi-do regions between October and December 2019, we enrolled 300 golfers with experience of golf travel to golf courses in Southeast Asia. To recruit subjects, we used purposive sampling, which is a non-probability sampling technique, and subjects were asked to complete a self-report questionnaire. All questions except those on demographic characteristics were rated on a 5-point Likert scale. After excluding 67 questionnaires with insincere or missing responses, the remaining 233 questionnaires were used in the analysis. The collected data were analyzed using AMOS 22.0 and SPSS 22.0. The general characteristics of the subjects for the quantitative study are presented in Table 3.

#### 3.2.3. Operational Definitions of Variables and Composition of Measured Items

Referring to studies by Park [41], Spreng and Mackoy [26], Cracolici and Nijkamp [18], Cooper and Boniface [15], and Weaver and Opperman [42], the choice properties of golf courses were composed of the major factors compared and reviewed when selecting a golf course. Referring to studies by Elsharnouby [43], Hutchinson et al. [31], Hansemark and Albinsson [44], Petrick [30], Dick and Basu [45], and Oliver [25], customer satisfaction was defined as the positive emotions formed after actual experience of the golf course. Based on studies by Zeelenberg and Pieters [36], Zeithaml and Bitner [46], Brady et al. [34], Garbarino and Johnson [37], and Berry and Parasuraman [38], intention to revisit was defined as an intention to repeatedly use the same product or service. The measured items originally consisted of 32 questions on the choice attributes of golf courses (6 on course layout, 4 on course management, 4 on food, 3 on practice facilities, 4 on price, 5 on operations management, 3 on accessibility, and 3 on lodging), 10 questions on customer satisfaction, and 6 questions on intention to revisit. To test the validity of the survey instrument in this study, we performed confirmatory factor analysis and reliability analysis. All questions that did not meet the criteria were deleted before statistical analysis. Table 4 shows the results of the validity and reliability analysis for choice attributes. Based on the results of the confirmatory factor analysis, 30 out of the original 32 questions were used in the final analysis. Table 5 shows the results of the validity and reliability analysis for customer satisfaction; 8 out of the original 10 questions were used in the final analysis. Table 6 shows the results of the confirmatory factor analysis for the intention to revisit; 4 out of the original 6 questions were used in the final analysis.

### 3.3. Qualitative Study

The qualitative study aimed to analyze the main determining factors for Korean golfers’ choices of golf courses in Southeast Asia. Hence, we directly visited three golf courses located in Malaysia over two months between December 2019 and February 2020, and conducted 16 in-depth interviews at a rate of 2 interviews per week for 8 weeks. The data obtained from this qualitative study were then compared with the contents of the quantitative study.

There were 13 participants in the in-depth interviews for this qualitative study, consisting of 1 person affiliated with a golf travel agency, 2 persons managing golf courses, 2 professional golfers, and 8 golf travelers. The general characteristics of the participants in the qualitative study are shown in Table 7. Using semi-structured and unstructured interviews, we explored the effects of the choice attributes of Southeast Asian golf courses on customer satisfaction and repeat visits. We focused on drawing out the real opinions of the participants in the in-depth interviews [47]. To ensure the ethicality of this study, before obtaining consent from the participants, we thoroughly explained the objectives and content of the study, and how the results would be used. During the in-depth interview, we obtained consent to record the interview. We obtained prior consent under the condition that all the contents of the interview would be anonymized, and that no identifiable information would be revealed. We also explained to participants that they did not have to mention anything during the interview that made them uncomfortable or that they did not want revealed, and that they could refuse to participate in the study [47].

## 4. Results

### 4.1. Quantitative Study Results

#### 4.1.1. Testing Construct Validity

To analyze the effects of choice attributes of golf courses in Southeast Asia on customer satisfaction and intent to revisit, we analyzed the construct validity of variables extracted through factor analysis. We derived the construct validity and AVE to analyze convergent validity. The construct validity should be ≥0.7 to satisfy convergent validity, and the AVE should be larger than the square of the correlation coefficients with each factor to satisfy discriminant validity [48]. In this study, all factors confirmed construct validity. The results of the correlation analysis are shown in Table 8.

#### 4.1.2. Hypothesis Testing

##### Testing H1 and H2

To analyze the effects of Southeast Asian golf course choice attributes on customer satisfaction and intention to revisit, path analysis was used to test the research hypotheses. From “H1. Golf course choice attributes have a positive effect on customer satisfaction,” course management, price, operations management, and lodgings showed positive effects on customer satisfaction. Course layout, food, practice facilities, and accessibility did not show a positive effect on customer satisfaction. “H2. Customer satisfaction has a positive effect on intention to revisit” was accepted. Table 9 shows the results of testing H1 and H2.

##### Testing H3

**Figure 2:**
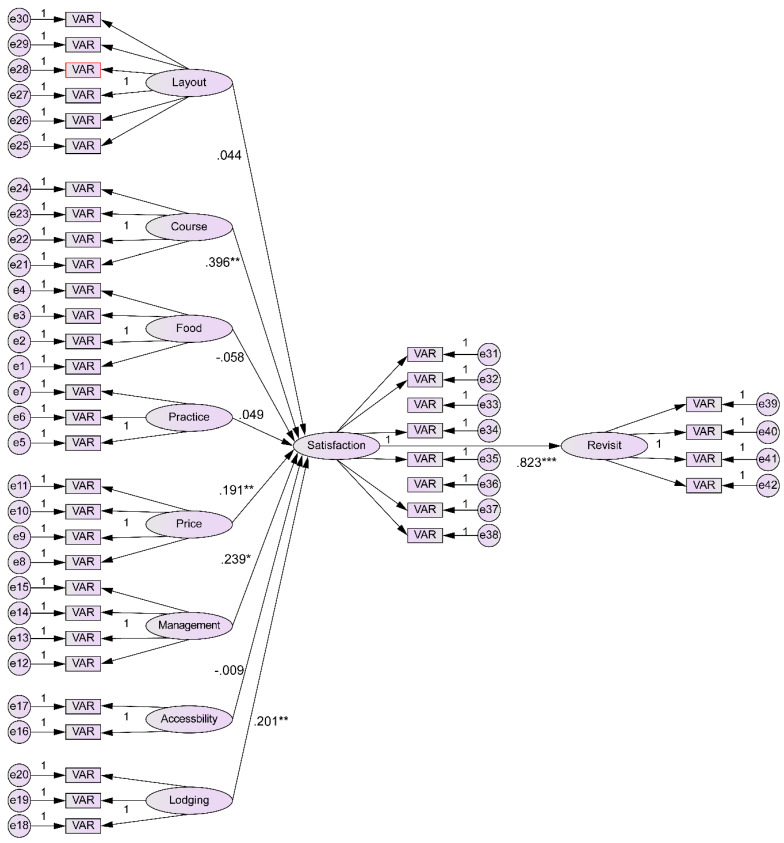
Validated model.

To test the mediation effect of customer satisfaction on the relationship between intention to revisit and the choice attributes of golf courses in Southeast Asia, we used a bootstrap technique to analyze the mediation effect of customer satisfaction in H3. Based on the results, among the subcategories of choice attributes in “H3. Customer satisfaction has a mediation effect on the relationship between intention to revisit and golf course choice attributes,” a mediation effect of customer satisfaction was only observed in the relationships of intention to revisit with course management and price. There was no mediation effect on the relationships of intention to revisit with course layout, food, practice facilities, operations management, accessibility, and lodgings. Table 10 shows the results of testing H3. The validated model for this study is shown in Figure 2.

### 4.2. Qualitative Study Results

The qualitative study aimed to empirically analyze the key factors determining Korean golfers’ choice of golf courses in Southeast Asia, and to compare the results with the quantitative analysis. Before conducting the qualitative study, through a meeting with three experts in related fields, we decided that it would be best to subcategorize the results according to the length of travel. After setting the two factors of short-term and long-term stays, eight golf course choice attributes used in the quantitative study (course layout, course management, food, practice facilities, price, operations management, accessibility, and lodgings) were taken as subfactors in each subcategory for comparison with the quantitative results. This content is summarized in Table 11.

#### 4.2.1. Short-Term Stay

In this study, we defined golfers who stayed for ≤7 days in a Southeast Asian golf course as short-term stays. We conducted in-depth interviews on golf course choice attributes with related workers and travelers who had participated in short-term golf travel. The interview results are shown below:

“I travel overseas for golf every winter. Because I have to go to work, I cannot stay longer than one week, and so the thing I am most interested in is being able to play as many rounds as possible while I am here. I like a place that has direct flights, so it’s easy to get to, and where I can play unlimited rounds. Even if the course has good accessibility, if there are too many other teams, or the games take too long due to lack of proper management, it limits the amount of golf I can play. In that case, even if the other facilities are excellent, I will not visit that course again.”

(Golf traveler J)

“I like somewhere that offers not only golf, but also opportunities for relaxation and tourism. Even if I do not play a lot of golf, I think the quality of the accommodation and the food are important. I want a place that is close to famous tourist sites, so that I can do some sightseeing as well. I do not mind if it’s a little expensive, as long as the accommodation, clubhouse, and restaurants are clean, and there are amenities such as a swimming pool and a gym, so that I can rest comfortably. Because I am only staying for a short time, I want somewhere with less travel time.”

(Golf traveler K)

“We have a golf group at school, so we travel to Southeast Asia for golf in the winter. Korea is too cold to play golf properly in the winter, but also, the golf courses are too expensive, so I usually go to a driving range or screen golf center with other group members. I prefer the golf courses in Southeast Asia because they’re less expensive. While I am here, I try to get in as many rounds as possible, but that is only possible if the course manages the flow of matches to prevent congestion. It’s even better if the course has good accessibility, so that I can get some golf in on the days I arrive and leave.”

(Golf traveler L)

“I started traveling every winter with a pro to improve my golf ability. Because I usually just go wherever the pro golfer decides, I have not given too much thought to the location. Still, I think the most important factors are keeping the putting greens fast to ensure the proper conditions for golf and good course management to prevent congestion.”

(Golf traveler M)

“As a professional golfer, I am currently giving golf lessons at an outdoor practice range in Korea. However, in winter, it’s too cold to give lessons in Korea, so I hold a golf camp in Southeast Asia, which is relatively inexpensive. Personally, I usually run a golf camp for short-term participants, lasting 4–5 days; the guests typically prefer a location with direct flights to shorten the travel time, and with good course management to prevent congestion and allow for rounds to be played comfortably. For me to conduct lessons, the course needs to have a practice range and practice greens to allow putting and short game practice, and so practice facilities are essential for the golf camp.”

(Professional golfer F)

“For golf travel, I think it’s crucial to distinguish between short-term and long-term travel. Short-term golf travel is usually like a holiday or sightseeing, while long-term golf travel typically has a clearer purpose. For short-term stays, travelers mostly use a travel agency like ours to make reservations, depending heavily on travel agency staff residing at the golf course. On the other hand, long-term travelers are mostly referred through professional golfers, and so the dependence on professional golfers is higher.”

(Golf travel agency affiliate A)

“Our golf course mostly caters to short-term customers. Compared to nearby competitors, we tend to have more guests. Our base price is inexpensive, we allow unlimited rounds, and we have a pricing system that offers several options, allowing customers to choose according to their needs. We sell a relatively cheap membership, and members can bring up to 3 guests, who all pay the same price as the member. We believe that this system is the reason why we have more guests than nearby competitors. Most of the golf-lovers who visit Southeast Asia just want to play a lot of rounds at a cheap price.”

(Y golf course manager C)

Based on the above interviews, short-stay golfers visiting golf courses in Southeast Asia considered accessibility, especially travel time, to be very important. This appears to be because of the short duration of the holiday, meaning that the travelers were sensitive to losing potential golfing time because of longer travel times. Golf travelers J, K, and L and Professional golfer F mentioned the importance of accessibility, whereas the statements of Golf travelers J and L, Professional golfer F, and Y golf course manager C showed that inexpensive price and unlimited rounds were extremely important considerations. Moreover, the interviews with Golf travelers J, L, and M and Professional golfer F showed that professional management, including tee-off management and match progression, were closely related to being able to play more rounds, by controlling the flow of the golf course. People such as Golf traveler K, who were seeking rest and a holiday, were not greatly influenced by the price, operations management, or course management, but showed considerable interest in choice attributes such as lodgings, amenities, and food. However, people such as Golf traveler M, who visited the golf course to learn from a professional golfer, placed great importance on overall operations management as well as course management, including the speed of the greens. However, as mentioned by the Golf travel agency affiliate A, most short-stay travelers use a travel agency rather than visiting via a professional golfer. Thus, the major factors affecting the choice of golf course in Southeast Asia for short-term golf travelers were accessibility, price, and operations management. However, golfers who desired rest and a holiday were less sensitive to price while placing more weight on food and lodgings. Based on these results, short-term golf travel should implement more precise targeting of customers, differentiating between more and less eager golfers. As discussed by Y golf course manager C, the rate of revisits can be improved by targeting eager golfers with promotions offering membership at reasonable prices and discounted prices for accompanying guests.

#### 4.2.2. Long-Term Stay

Golfers who stayed in Southeast Asian golf courses for ≥7 days were defined as long-term stays. We conducted in-depth interviews on golf course choice attributes with related workers and travelers who had participated in long-term golf travel. The interview results are shown below:

“Our golf course does not have direct flights from Korea, and it’s not located in a famous tourist area, which makes it really difficult to entice golfers to come from overseas. Generally, the start of January to the end of February is the offseason for golf in Southeast Asia. Apart from a few golf courses with good accessibility, most golf courses are suffering difficulties in business. As a result, the number of golf courses becoming insolvent is growing each year. In response to this situation, our golf course has been targeting long-term stay golfers who come for practice, by expanding out practice ranges and practice facilities, and increasing the difficulty of the course. We have formed partnerships with professional golfers from Korea for this purpose.”

(X golf course manager B)

“As a professional golfer, I visit golf courses in Southeast Asia with other golfers every winter for preseason training. We typically stay for anywhere between 1 and 3 months. We are not especially concerned about the accessibility of the golf course. The most important factor is an environment that is suitable for training. The course should separate athletes from the general public to ensure that there are no problems with congestion of play. Since most golf athletes play much faster than the general public, expert management is essential. In addition, factors that can help improve performance are key, and so higher course difficulty and faster greens should be maintained. Because golf athletes cannot train in Korea in the winter, they stay overseas for long periods of time for training. For this reason, it is crucial to have plenty of practice ranges and practice greens.”

(Professional golfer, E)

“While long-stay golfers often go through professional golfers, there has been a gradual increase in the number of people, including retirees, using our golf travel agency [for long-stay golf travel]. Most of these people are traveling to a warm location during the cold winter, they often cook for themselves, and typically just play around 9 holes of golf per day. These customers are often sensitive to the price, and so the most important point is to construct an inexpensive package suited to a long stay including the minimum options. Nevertheless, because these customers like to bring other retired acquaintances, if they feel that the package composition and price are reasonable, it is possible to continually increase the number of visitors.”

(Golf travel agency affiliate A)

“I am the mother of a student athlete. We usually just follow wherever the pro decides to go, but the pro tends to consider the opinions of the parents when selecting the location. It’s expensive to send your child to preseason training—the costs include board and living fees for my child and I, airplane tickets, lesson fees, and shared costs between all the students—so price is what we think about most. The next most important factors after cost are food and dining facilities, because we have to stay there for a long time, and suitable practice facilities for training. It’s better if the lodgings, golf course, and practice range are close together, so there’s less distance to travel.”

(Golf traveler J)

“I am a golf athlete at high school. I’ve been traveling overseas for preseason training every winter since elementary school. The location is decided by the pro who’s teaching me and I follow along. I’ve been to several places so far, but most of the preseason training locations in Southeast Asia are better for training than those in the US or Australia, because the course, practice range, accommodation, and dining facilities are close together. In the US, the courses were nice, but the accommodation was too far, and so I only ate hamburgers or fast food for lunch every day. The pro tends to consider the opinions of the students and parents when deciding the location for training, but personally, I think the most important factors are the food, practice facilities, and fast greens.”

(Golf traveler H)

“I practice a lot in spring, summer, and fall to get better at golf, but in winter, it’s hard to practice because it’s too cold. The pro I learn from holds an overseas camp every winter where they teach golfers, and I’ve been taking part as well. The location is decided by the pro, but as long as the greens are kept fast and the course is managed to prevent congestion, I do not have any complaints. However, actually, I do not think I would come by myself if it was not for the lessons with the pro.”

(Golf traveler G)

“After retirement, I’ve been coming to Southeast Asia every summer and winter for 2 months at a time. I do not actually play golf every day, and I usually make my own food. As a result, I like a course with a reasonable price for a long stay, where the lodgings have facilities for me to make my own food, and where there is a community of similarly aged people that I can socialize with.”

(Golf traveler N)

**Figure 3:**
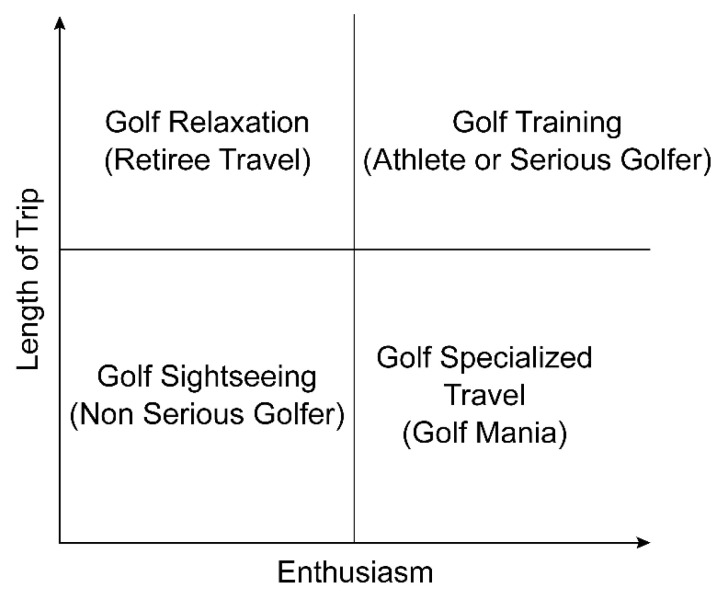
Golf travel matrix.

Based on the above interviews, the important factors for long-stay golfers visiting Southeast Asian golf courses were expert operations management to control the tee-offs and the flow of players through the course, course management to maintain fast greens, practice ranges, and practice facilities for short games and putting, and, since guests are staying for a long period of time, diverse options for food. The main characteristic of long stays was the major role played by professional golfers. X golf course manager B reported that because they formed partnerships with capable Korean professional golfers, they were able to ensure a steady flow of visitors in the offseason despite poor accessibility due to the absence of direct flights. This content shows that most long-stay golfers are strongly connected with professional golfers. General golfers, such as golf traveler G, also come to receive lessons, whereas athletes, such as golf traveler H, and the parents of athletes, such as golf traveler J, were both brought along by professional golfers. In these cases, we found that the location was decided by the professional golfer. As another type of long-term stay, there is a growing trend for long-term golf package stays via a local travel agency, typically used by retirees, as mentioned by golf travel agency affiliate A. Thus, long-term stays need to be divided broadly into two types: golf training and retiree trips. To summarize, for golf training, groups of guests form around a professional golfer, and key choice attributes are operations management, course management, and practice facilities, whereas price and food are also considered. However, for retiree trips, as mentioned by golf traveler N and golf travel agency affiliate A, it is important to have a flexible pricing structure, offering various options for the frequency of golf and whether or not meals will be provided. Moreover, if a community is formed to allow retirees to socialize together, we believe it will be much easier to promote retiree trips. When we analyzed the factors of short and long stays, considering the length of stay and golfer enthusiasm, we constructed a matrix, as shown in Figure 3, indicating the extent of participation in golf travel.

## 5. Discussion

In this discussion, we compare the results of our quantitative study with those of previous studies, following the order of the research hypotheses, and conduct an empirical analysis by comparing the choice-determining factors for golf courses identified in the qualitative study. The quantitative study investigated the relationships between the choice attributes of golf courses in Southeast Asia (course layout, course management, food, practice facilities, price, operations management, accessibility, lodgings), customer satisfaction, and intention to revisit, and analyzed the role of customer satisfaction in the relationship between choice attributes and intention to revisit. In the qualitative study, we conducted in-depth interviews with participants to identify the major factors determining choices of golf courses in Southeast Asia depending on the participant and their goals. Based on these results, the following discussion points are as follows.

First, “H1. Golf course choice attributes will have a positive effect on customer satisfaction” was partially accepted. Among the choice attributes, course management, price, operations management, and lodgings were found to have an effect on customer satisfaction. Course layout, food, practice facilities, and accessibility did not show any effect on customer satisfaction. These results are supported by a study by Petrick et al. [19], in which the choice attributes of course management and operations management related to game progression have strong effects on golfer satisfaction. In a study by Won et al. [21], course management was identified as the most important factor in users’ choice of golf course, which is similar to our findings. Thus, course management and operations management are key choice attributes for golf courses. However, given that each golfer has their own tendencies and purpose for visiting the golf course, the preferred choice factors will also differ between golfers. Therefore, to analyze golfers who visited golf courses in Southeast Asia in more detail, we subcategorized golfers according to the length of their golf travel and investigated choice-determining factors for short-stay and long-stay golf travelers. First, we found that accessibility, price, and operations management were the key choice factors for short-term stays. Moreover, for golf travelers looking for rest and tourism more than golf, food and lodgings also affected the choice of golf course. Meanwhile, operations management, course management, and practice facilities were major choice factors for long-term stays, and price and food also had an effect. When we compared our qualitative study with our quantitative study, the results were mostly consistent. The one difference in the qualitative study was that the choice factors included accessibility for short-term stays and practice facilities for long-term stays. Thus, one characteristic of short stays is that, since the customer is traveling to play golf, they want to minimize the time wasted on traveling. For long stays, because the aim is to practice and improve golf ability, practice facilities are important.

Second, “H2. Customer satisfaction has a positive effect on intention to revisit” was accepted. This is similar to the findings in a study by Petrick and Backman [20] who analyzed the causal relationship between guest satisfaction and revisits due to increasing competition among golf courses. Analogously, in a study by Petrick et al. [19], a satisfactory experience leads to an increase in revisits. The results of our study indicate that customer satisfaction among golfers at golf courses in Southeast Asia is linked to customer behaviors such as revisits and positive recommendations. However, because the preferred choice attributes differ depending on the golfer and their objectives, it is important to first understand the client.

Third, “H3. Customer satisfaction has a mediation effect on the relationship between intention to revisit and golf course choice attributes” was partially accepted. Among the choice attributes for golf courses, only customer satisfaction showed a mediation effect in the relationships of intention to revisit with course management and price. In this study, course management, price, operations management, and lodgings did not affect customer satisfaction. Operations management and lodgings did affect customer satisfaction, but we can surmise that there was no effect on revisits because of the role of customer satisfaction as a mediator. Meanwhile, course management and price were found to affect revisits via customer satisfaction as a mediator. Our results are supported by studies by Petrick et al. [19] and Won et al. [21], which also showed that course management is an important factor affecting revisits. Our qualitative study also showed that for short-term specialized golf travel and long-term golf training, which show a high level of involvement in golf, course management and price were major determining factors in golfers’ choice of golf courses in Southeast Asia. Conversely, for short-term golf sightseeing and long-term golf relaxation, course management was not a major determining factor, and price was only a determining factor for long-term golf relaxation. Thus, among travelers with a high level of involvement or who attributed a high degree of importance to golf, the results were largely consistent with the quantitative study, and for travelers with less involvement in growth, golf-related factors had less of an effect on the choice of location or likelihood of revisits.

## 6. Conclusions

The purpose of this study was to understand how the customer behavior of Korean golf travelers in Southeast Asia is affected by the choice attributes of golf courses, through empirical analysis. To this end, using the triangulation design of Creswell and Clark [40] as a type of mixed-methods research, we compared the results of quantitative study and qualitative study. Based on these research results, we suggest the following managerial implications and proposals.

### 6.1. Managerial Implications

#### 6.1.1. Short-Term Stays

In this study, as shown in Figure 3, short-term stays were divided into based on the results, specialized golf travel and golf sightseeing. Specialized golf travel refers to people whose travel itinerary revolves only around golf, whereas golf sightseeing refers to people who seek other activities along with golf. The major choice-determining factors for specialized golf travel are accessibility, price, and operations management. Most of the guests are people hoping to play golf intensively for a short period, at a low cost because it is not possible to play golf in Korea in winter. To minimize movement time and play the maximum number of rounds in a limited time, professional operations management of the golf course is essential. However, travelers for golf sightseeing, who aim to combine golf with tourism or rest, are much more concerned with lodgings and food. Therefore, when targeting short-stay golfers, it is essential to differentiate preferred choice factors depending on the golfer’s degree of involvement.

#### 6.1.2. Long-Term Stays

As shown in Figure 3, long-term stays were divided into golf training and golf relaxation based on the results. Golf training refers to athletes or serious golfers with a high level of investment who stay long-term to improve their golf ability. Golf relaxation refers to people who are not traveling only for golf, but want to relax in a warm environment while also enjoying golf, and this includes retirees. For golf training, the main choice-determining factors were operations management, course management, and practice facilities, whereas price and food also need to be considered. Because golf training involves long-term stays, centered around professional golfers, to learn and train for improving golf ability, it is essential to ensure that players move through the course smoothly through professional operations, and to maintain consistent fast greens to improve technical skills. Moreover, practice ranges, putting greens to practice putting, and practice facilities to practice approach and bunker shots are extremely important for lessons and training. Usually, it is difficult to practice properly in the cold winter, and hence players move to a warmer region to maintain their touch and continue training. Thus, we can surmise that the cost of training and a proper diet to maintain nutrition are also important. For golf relaxation, an efficient pricing system with diverse options according to golfing frequency and meal requirements is an important determining factor. A growing number of retirees seek golf courses in Southeast Asia to enjoy golf and relaxation at a relatively low cost for a high quality of life. Hence, forming a community for retirees in the same cultural sphere is a key factor for increasing the number of these guests.

### 6.2. Proposals

From this study, we can infer that there might be differences in customer behaviors compared to golfers visiting domestic golf courses or visiting other regions overseas. Therefore, one limitation of our study is that the results cannot be considered representative of all overseas golf travel. We propose the following future studies.

First, based on our qualitative analysis, we classified golf travel to Southeast Asia into four categories: specialized golf travel and golf sightseeing for short-term stays, and golf training and golf relaxation for long-term stays. We propose that future research differentiating participants according to their aims, location, and other characteristics should be conducted. Research on specific subdivisions of golfers is expected to produce empirical results that can be applied to the sports industry.

Second, there is a growing trend for overseas golf travel to China and Japan outside of the winter season. We believe that very useful data could be acquired by conducting detailed analysis of non-winter golf travel in nearby countries outside of Southeast Asia, differentiating between different types of travel, purpose of travel, and methods of golf course operation.

Third, golf courses provide intangible service-based products to enable golfing competition. Therefore, there is a need for research to test and compare service quality at golf courses, differentiating between golf-related and non-golf-related factors.

Finally, in this study, we investigated golf travel, but useful empirical results could also be obtained by conducting qualitative or mixed-methods research on travel for other sports, differentiating between travel for travel’s sake, or for improving skill.

## Figures and Tables

**Table 1:** Current state of the golfing population.

	2007	2012	2014	2017
Domestic golfing population (million)	2.75	4.70	6.19	7.61
Annual golf expenditure (trillion KRW)	12.9516	23.976	25.4880	25.1856
Overseas golf tourism population (million)	0.67	1.65	2.05	2.64
Mean overseas golf expenditure per person (million KRW)	1.82	1.75	1.90	1.41

Source: 2018, Korea Golf Index [9].

**Table 2:** Overseas golf travel destinations (2018).

Thailand	20.3%	Malaysia	5.1%	United States	4.4%
Vietnam	14.3%	Singapore	3.7%	Australia	4.5%
Philippines	12.8%	Japan	13.2%	New Zealand	3.9%
Indonesia	5.1%	China	9.6%	Other	3.1%

Source: 2018, Korea Golf Index [9].

**Table 3:** Subjects’ general characteristics.

Variable	Category	Frequency (Persons)	Percentage (%)
Gender	Male	133	57.1
	Female	100	42.9
Age (years)	<30	51	21.9
	30–39	54	23.2
	40–49	64	27.5
	≥50	64	27.5
Golf experience (years)	<1	42	18.0
	≥1 and <3	58	24.9
	≥3 and <5	53	22.7
	≥5	80	34.4
Average score	≤81	59	25.3
	82–90	48	20.6
	91–100	80	34.3
	≥101	46	19.7
Average length of stay per year (days)	<5	121	51.9
	≥5 and <15	81	34.7
	≥15	31	13.3
Occupation	Self-employed	29	12.4
	Homemaker	39	16.7
	Office worker	65	27.9
	Professional worker	40	17.2
	Golfer or golf instructor	40	17.2
	Retiree	20	8.5
Type of golf travel	Package from domestic travel agent	124	53.2
	Package from local travel agent	84	36.1
	Direct reservation with the golf course	14	6.0
	Membership with the golf course	11	4.7
Purpose of golf travel	Golf tourism	86	36.9
	Preseason training	35	15.0
	Golf lessons	35	15.0
	Family holiday	19	8.2
	Business	19	8.2
	Rest	39	16.7
Golf travel destination (Southeast Asia)	Thailand	94	40.3
	Vietnam	51	21.9
	Philippines	39	16.7
	Malaysia	27	11.6
	Indonesia	9	3.9
	Other	13	5.6
Total		233	100

**Table 4:** The results of validity and reliability analysis for choice attributes.

Factor	Variable	Standardized Coefficient	S.E.	C.R.	*p*	Reliability
Layout	Course (arrangement, combination, and variability of short, middle, and long holes)	0.681				0.837
	Course (hole width and length)	0.694	0.109	9.279	***	
	Course (relationships between adjacent holes, e.g., safety, ease of transport)	0.698	0.116	9.316	***	
	Course (variability in the elevation and locations of holes)	0.674	0.119	9.037	***	
	Tactical aspects of the course (locations of hazards, bunkers, and obstacles)	0.708	0.111	9.440	***	
	Scenery surrounding the course (beauty and harmony)	0.645	0.129	8.693	***	
Course management	Maintenance and management of the teeing grounds	0.692				0.809
	Maintenance and management of the fairways	0.727	0.101	9.727	***	
	Maintenance and management of the greens	0.748	0.106	9.962	***	
	State of the sand in bunkers and cleanliness of the hazards	0.704	0.104	9.463	***	
Food	Taste and quality of food	0.759				0.844
	Menu diversity	0.737	0.100	10.809	***	
	Atmosphere of restaurants	0.799	0.092	11.692	***	
	Convenience of use of restaurants	0.743	0.087	10.905	***	
Practice facilities	Driving range	0.655				0.758
	Short game practice area (approach and bunkers)	0.794	0.133	9.138	***	
	Putting practice area	0.721	0.118	8.688	***	
Price	Golf cost	0.716				0.847
	Food cost	0.801	0.108	11.133	***	
	Lodging cost	0.853	0.118	11.647	***	
	Cost of other facilities	0.687	0.106	9.657	***	
Operations management	Reliability of golf course management	0.777				0.829
	Expertise of management staff	0.709	0.093	10.633	***	
	Golf course flow management (including tee-off time control)	0.721	0.100	10.821	***	
	Attitude and service of staff, including the clubhouse, restaurants, and lodging	0.769	0.089	11.592	***	
Accessibility	Flight time to the destination airport	0.905				0.834
	Transit time from the destination airport to the golf course	0.791	0.088	10.451	***	
Lodging	State of maintenance for beds, air conditioning, etc. in the lodgings	0.776				0.854
	Comfort and cleanliness of the bathrooms/toilets in the lodgings	0.852	0.085	12.942	***	
	State of cleanliness of the lodgings (housekeeping)	0.818	0.087	12.525	***	
χ^2^ = 673.712; df = 377; *p* = 0.000; CMIN/df = 1.787; CFI = 0.913; GFI = 0.844; NFI = 0.825; RMSEA = 0.058; RMR = 0.028; *** *p* < 0.001.						

**Table 5:** The results of validity and reliability analysis for customer satisfaction.

Factor	Variable	Standardized Coefficient	S.E.	C.R.	*p*	Reliability
Customer satisfaction	I am satisfied with the prices provided by this golf course.	0.644				0.902
	I am satisfied with the way that this golf course responds to customer complaints.	0.664	0.125	8.794	***	
	I am satisfied with the staff working at this golf course.	0.710	0.126	9.288	***	
	I typically enjoy using this golf course.	0.786	0.123	10.066	***	
	I think this golf course is nice to use.	0.775	0.130	9.965	***	
	I am satisfied with the services and facilities provided by this golf course.	0.697	0.125	9.155	***	
	I am very satisfied with my relationship with this golf course.	0.773	0.136	9.943	***	
	I am very satisfied with this golf course.	0.801	0.130	10.212	***	
χ^2^ = 56.010; df = 20; *p* = 0.000; CMIN/df = 2.800; CFI = 0.962; GFI = 0.943; NFI = 0.942; RMSEA = 0.088; RMR = 0.019; *** *p* < 0.001.						

**Table 6:** The results of validity and reliability analysis for intention to revisit.

Factor	Variable	Standardized Coefficient	S.E.	C.R.	*p*	Reliability
Intention to revisit	I will continue to use this golf course as a destination for overseas golf travel.	0.830				0.884
	When I choose an overseas golf course, I will choose this course first.	0.858	0.076	14.688	***	
	I am likely to revisit this golf course.	0.782	0.074	13.161	***	
	I intend to recommend this golf course to people I know as a destination for overseas golf travel.	0.768	0.070	12.861	***	
χ^2^ = 1.602; df = 2; *p* = 0.449; CMIN/df = 0.801; CFI = 1.000; GFI = 0.997; NFI = 0.997; RMSEA = 0.000; RMR = 0.005 *** *p* < 0.001.						

**Table 7:** General characteristics of the qualitative study participants.

Participant	Gender	Age	Reason for Stay	Occupation
1. Golf travel agency affiliate A	M	55	Working for a related business	Golf travel agency director
2. X golf course manager B	M	51	Working for a related business	Golf course director
3. Y golf course manager C	M	45	Working for a related business	Worker for a golf course management agency
4. Professional golfer E	M	44	Working for a related business	Golf instructor
5. Professional golfer F	M	38	Working for a related business	Golf instructor
6. Golf traveler G	M	50	Long-term stay	Business
7. Golf traveler H	M	18	Long-term stay	Golfer
8. Golf traveler I	F	48	Long-term stay	Golfer’s parent
9. Golf traveler J	M	42	Short-term stay	Office worker
10. Golf traveler K	F	39	Short-term stay	Professional worker
11. Golf traveler L	M	26	Short-term stay	University student
12. Golf traveler M	M	59	Short-term stay	Professional worker
13. Golf traveler N	M	70	Long-term stay	Retiree

**Table 8:** The results of correlation analysis.

Factor	1	2	3	4	5	6	7	8	9	10
1	1									
2	0.544	1								
3	0.283	0.444	1							
4	0.331	0.287	0.328	1						
5	0.164	0.119	0.101	0.254	1					
6	0.429	0.467	0.242	0.279	0.300	1				
7	0.141	0.243	0.135	0.284	0.178	0.186	1			
8	0.302	0.312	0.259	0.272	0.130	0.376	0.297	1		
9	0.447	0.552	0.274	0.329	0.345	0.579	0.241	0.413	1	
10	0.338	0.442	0.247	0.231	0.169	0.375	0.217	0.341	0.667	1
Construct reliability	0.920	0.895	0.901	0.862	0.909	0.902	0.883	0.915	0.950	0.929
AVE	0.659	0.682	0.696	0.677	0.717	0.698	0.791	0.783	0.705	0.768
* The square of each correlation coefficient has been entered. 1. Course layout; 2. Course management; 3. Food; 4. Practice facilities; 5. Price; 6. Operations management; 7. Accessibility; 8. Lodgings; 9. Customer satisfaction; 10. Intention to revisit.										

**Table 9:** The results of testing H1 and H2.

Hypothesis	Path	Standardized Coefficient	S.E.	C.R.	*p*	Outcome
1-1	Course layout → Customer satisfaction	0.044	0.097	0.478	0.633	Rejected
1-2	Course management → Customer satisfaction	0.396	0.112	3.167	0.002 **	Accepted
1-3	Food → Customer satisfaction	−0.058	0.062	−0.726	0.468	Rejected
1-4	Practice facilities → Customer satisfaction	0.049	0.076	0.590	0.555	Rejected
1-5	Price → Customer satisfaction	0.191	0.060	2.807	0.005 **	Accepted
1-6	Operations management → Customer satisfaction	0.239	0.081	2.504	0.012 *	Accepted
1-7	Accessibility → Customer satisfaction	−0.009	0.044	−0.122	0.903	Rejected
1-8	Lodgings → Customer satisfaction	0.201	0.060	2.638	0.008 **	Accepted
2	Customer satisfaction → Intention to revisit	0.823	0.114	9.825	***	Accepted

* *p* < 0.05, ** *p* < 0.01, *** *p* < 0.001.

**Table 10:** Results for H3.

Hypothesis	Path	S.E.	Standardized Coefficient	Indirect Coefficient		*p*	Outcome
				Min	Max		
3-1	Course layout → Customer satisfaction → Intention to revisit	0.153	0.052	−0.187	0.211	0.797	Rejected
3-2	Course management → Customer satisfaction → Intention to revisit	0.186	0.397	0.081	0.656	0.015 *	Accepted
3-3	Food → Customer satisfaction → Intention to revisit	0.094	−0.051	−0.221	0.120	0.561	Rejected
3-4	Practice facilities → Customer trust → Intention to revisit	0.107	0.050	−0.137	0.213	0.645	Rejected
3-5	Price → Customer trust → Intention to revisit	0.081	0.190	0.034	0.295	0.021 *	Accepted
3-6	Operations management → Customer trust → Intention to revisit	0.166	0.228	−0.063	0.469	0.129	Rejected
3-7	Accessibility → Customer trust → Intention to revisit	0.072	−0.006	−0.185	0.139	0.904	Rejected
3-8	Lodgings → Customer trust → Intention to revisit	0.102	0.177	−0.011	0.352	0.052	Rejected

* *p* < 0.05, ** *p* < 0.01 *** *p* < 0.001.

**Table 11:** Subcategorization of golf course choice attributes for qualitative analysis.

Factor	Sub Factor
Short-term stay	Course layout
	Course management
	Food
	Practice facilities
	Price
	Operations management
	Accessibility
	Lodgings
Long-term stay	Course layout
	Course management
	Food
	Practice facilities
	Price
	Operations management
	Accessibility
	Lodgings

## Data Availability

Data sharing is not applicable to this article.
